# Impact of succussion on pharmaceutical preparations analyzed by means of patterns from evaporated droplets

**DOI:** 10.1038/s41598-019-57009-2

**Published:** 2020-01-17

**Authors:** Maria Olga Kokornaczyk, Sandra Würtenberger, Stephan Baumgartner

**Affiliations:** 1 Society for Cancer Research, Hiscia Institute, 4144 Arlesheim, Switzerland; 2 Scientific & Regulatory Affairs, Hevert-Arzneimittel GmbH & Co. KG, 55569 Nussbaum, Germany; 3 Institute of Integrative Medicine, University of Witten/Herdecke, 58313 Herdecke, Germany; 4 University of Bern, Institute of Complementary and Integrative Medicine, 3010 Bern, Switzerland

**Keywords:** Preclinical research, Materials science

## Abstract

The aim of the present study was to investigate if patterns obtained from evaporating droplets of pharmaceutical preparations reveal the impact of succussion on such medicinal products. For this purpose, five pharmaceutical preparations (*Echinacea* 10^−2^, *Baptisia* 10^−3^, *Baptisia* 10^−4^, *Luffa* 10^−4^, and *Spongia* 10^−6^) were prepared according to the European Pharmacopoeia guidelines for the production of homeopathic remedies, in three variants each: with varying numbers of succussion strokes (i) 100, (ii) 10 (succussed samples), and (iii) zero (gently mixed, unsuccussed sample). System stability was studied by means of systematic positive control experiments. Patterns were evaluated by means of computerized image analysis regarding grey level distribution, texture, and fractality. For all investigated pharmaceutical preparations, significant differences were found between the succussed and gently mixed samples; whereas, all three samples (prepared with 100, 10 and zero succussion strokes) could be significantly differentiated for *Luffa* 10^−4^ and *Spongia* 10^−6^ for one image evaluation parameter each. Control experiments showed a reasonable stability of the experimental set-up.

## Introduction

It is known that shaking a solution may have impact on proteins it contains^1,2^; the introduction of air bubbles into the solution^3^, as also the action of sharing forces, may trigger oxidation processes and aggregation of these molecules^1,4–6^. Solely, an accidental dropping of a vial has been reported to modify some proteins in suspension^2^. In pharmaceutical preparations, in some cases shaking and the thereby induced aggregation of proteins may influence their properties; therefore, the development of measures mitigating the shaking influence, like for instance development of new coatings for pre-filled syringes, is important and is addressed in recent investigations^6^.

The impact of agitation upon liquid pharmaceutical products has been investigated by means of various analytical approaches, including methods analyzing the particle formation (micro-flow imaging, dynamic light scattering, light obscuration method), protein degradation (size exclusion chromatography, tryptic digestion/HPLC), formation of free radicals (hydroxyphenyl fluorescein assay), and flow dynamics occurring during agitation (high speed imaging). Furthermore, different spectroscopy methods (fluorescence spectroscopy, Fourier transform infrared spectroscopy) and calorimetric methods (differential scanning calorimetry) have been applied for accessing the characteristics of agitated samples^1–6^. Here we propose for the first time to apply the droplet evaporation method (DEM) to access the characteristics of agitated pharmaceutical preparations in a comparably quick and integral manner.

Recently, methods based on droplet evaporation find application in various fields of science and technology, as for instance in fabrication of novel materials, microelectronics, ink-jet printing, coating technologies, bioassay manufacturing, condensation of solutes^7–9^, and also for analytical purposes. Among DEM’s analytical applications the most studied one is medical diagnosis^9,10^. It is based on the idea that in the case of some diseases patterns formed in desiccated droplets of some specific corporal fluids (e.g. blood, serum, tears, sweat) would differ depending on whether the fluid was taken from a diseased or healthy donor, since the disease would specifically modify the composition of the fluid.

In a previous study^11^, we have proposed DEM as a tool for a phenomenological, multi-factorial characterization of pharmaceutical preparations in a low dilution range (10^−2^-10^−6^). The corresponding experimental procedure consists in the evaporation of droplets of the diluted pharmaceutical preparations under controlled conditions, the consecutive inspection of patterns formed in droplet residues under an optical microscope with dark-field, and computerized image evaluation. In the present study further investigations by means of the same experimental protocol were conducted to determine, if it is possible to ‘visualize’ through the formation of self-assembled patterns any differences between succussed and unsuccussed samples; and furthermore, if the number of succussion strokes (N_S_) performed would show any impact on the patterns.

We have chosen to investigate the impact of shaking on pharmaceutical products according to the guidelines for homeopathic preparations, since the application of succussion is a mandatory procedure according to the European Pharmacopoeia^12^. The corresponding processing of pharmaceutical preparations from a given liquid substance consists in subsequent dilution steps (in a defined dilution ratio), each followed by succussion (i.e. introduction of some kind of motion into the liquid, mostly vigorous).

The choice of the pharmaceutical preparations was based on both their pattern forming properties (dendrite formation was preferably chosen)^11^ and their presence in the product *Sinusitis Hevert SL*. We investigated five different pharmaceutical preparations of vegetal (*Echinacea* 10^−2^, *Baptisia* 10^−3^, *Baptisia* 10^−4^, *Luffa* 10^−4^) and animal (*Spongia* 10^−6^) origin, prepared in three different variants each: succussed by the application of 100 or 10 strokes (succussed samples), or without succussion (only gently mixed control sample). The agitation technique applied was adopted from the production protocol as used by the pharmaceutical company Hevert-Arzneimittel GmbH & Co.

A crucial point in analytical methods involving images as main experimental output is the image evaluation and the choice of proper evaluation tools and evaluation criteria or parameters. In many studies DEM images were analyzed exclusively by means of visual evaluation^13^; despite the fact that the human eye is the most precise tool for form recognition, the visual evaluation of patterns may be subjective and it also strongly restricts the size of the image database to be evaluated. In previous studies we introduced the computerized measurement of several image evaluation parameters characterizing the images in terms of their grey level distribution, texture^11^, and fractality^14^. The parameter *grey level distribution* measures the image brightness^15^, which in case of DEM images provides information on the structures size, thickness of branches, and their brightness. The size of the structure can be assessed in a more precise way by means of the parameter *foreground pixel*, which measures the structure’s area^16^, however does not access the brightness. The parameter *entropy* is an attribute of the grey level co-occurrence matrix measuring how often different pixel brightness values occur in an image; in particular, *entropy* characterizes the heterogeneity of the brightness values distribution and describes so the image’s disorder^17^. Finally, the parameter *local connected fractal dimension* measures the fractal dimension of structures in a pre-defined size range and accesses so the structures complexity^16^. Moreover, in the present study we added the parameter *lacunarity*, a complementary measure to fractal dimension, characterizing the gaps in-between the structure elements^16^ and providing so information about the structure’s density.

## Results

### Qualitative description of the patterns

**Figure 1:**
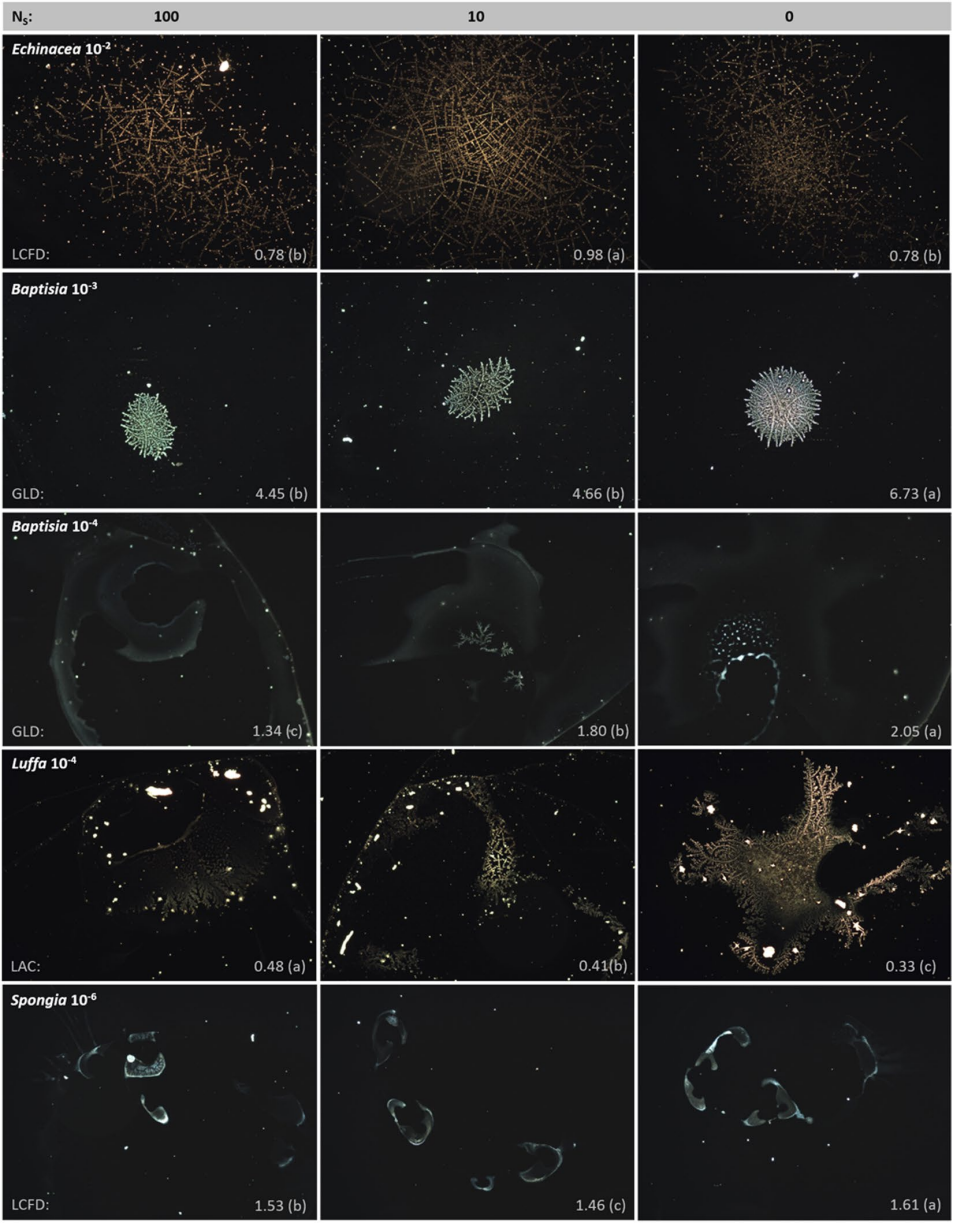
Examples of patterns formed in evaporating droplets of liquid pharmaceutical preparations; the droplet residues dried on a glass substrate were photographed by means of dark-field microscopy in magnification 100×. In rows patterns obtained from *Echinacea* 10^−2^, *Baptisia* 10^−3^, *Baptisia* 10^−4^, *Luffa* 10^−4^, and *Spongia* 10^−6^ are represented, whereas in columns varies the preparation manner consisting in the performance of vertical succussion strokes (N_*S*_ = 100, 10, or 0). Pattern examples derive from main experiments (cf. Fig. 2) and were selected based on an image-analysis parameter value (*grey-level distribution* (GLD), *lacunarity* (LAC), and *local connected fractal dimension* (LCFD)), which is close to the mean value of the corresponding parameter. Different letter codes (a, b, c) are significantly different (p < 0.05).

When analyzed by means of DEM, the five here investigated pharmaceutical preparations created visually recognizable and easily identifiable patterns (Fig. 1). In case of *Echinacea* 10^−2^, *Baptisia* 10^−3^, and *Luffa* 10^−4^ the patterns consisted of dendritic, fractal-like structures placed in the droplet center. *Echinacea* 10^−2^ created large, dense networks of very fine ramifications, *Baptisia* 10^−3^ created rather small, roundly shaped structures, and *Luffa* 10^−4^ structures made out of rather few and thick dendrites. *Baptisia* 10^−4^ created unspecific patterns consisting of lines, smears, and, in some cases, single dendrites distributed all over the droplet. Whereas, *Spongia* 10^−6^ created one to five filled, wavy forms per droplet, characterized by a concave and a convex side, placed near to each other and facing each other with the concave sides.

In general, in all pharmaceutical preparations, the impact of succussion on the patterns was visually perceptible in a varying, but rather small degree, and it seemed to decrease the structure’s ordering.

### Computerized pattern evaluation

The results of the computerized pattern evaluation of the pharmaceutical preparations produced with different numbers of succussion strokes (N_S_ = 100, 10, 0), the corresponding systematic positive control experiments, and the F-tests of the analysis of variance for *Echinacea* 10^−2^, *Baptisia* 10^−3^, *Baptisia* 10^−4^, *Luffa* 10^−4^, and *Spongia* 10^−6^ are shown in Tables 1–5, respectively.

**Table 1:** Results of pattern evaluation of *Echinacea* 10^−2^ samples prepared with different numbers of succussion strokes (N_S_ = 100, 10, or 0) and systematic positive control (SPC) experiments (on the left) and F-test of the two-way analysis of variance for the factors *N*_*S*_ and *day* (on the right). Mean values with different letter codes (a, b, c) are significantly different (p < 0.05).

	*Echinacea* 10^−2^			SPC			Factor	*Echinacea* 10^−2^		SPC	
	N_S_	N	Mean	N_S_	N	Mean		*F*	*p*	*F*	p
GLD	100 10 0	138 127 134	22.28 a 22.11 a 20.63 b	10 10 10	140 133 133	7.78 a 8.23 a 7.54 a	N_S_ 133 Interaction	6.25 81.77 2.15	0.0021** <0.0001*** 0.0740 ns	1.40 3.00 2.64	0.3694 ns 0.0515 ns 0.0332*
Entropy	100 100	138 127 134	3.74 a 3.72 a 3.48 b	10 10 10	140 130 133	3.11 a 3.13 a 3.04 a	N_S_ Day Interaction	3.05 119.19 1.39	0.0484* <0.0001*** 0.2369 ns	0.38 17.17 3.43	0.6840 ns <0.0001*** 0.0089**
FP	100 10 0	113 105 113	8.58 × 10^3^ a 9.96 × 10^3^ a 8.23 × 10^3^ a	10 10 10	140 133 133	6.20 × 10^3^ a 6.68 × 10^3^ a 5.64 × 10^3^ a	N_S_ Day Interaction	2.02 68.20 2.66	0.1348 ns <0.0001*** 0.0327*	1.12 21.18 1.88	0.3281 ns <0.0001*** 0.1137 ns
LCFD	100 10 0	113 105 113	0.79 b 0.94 a 0.78 b	10 10 10	140 133 133	0.61 a 0.64 a 0.60 a	N_S_ Day Interaction	6.47 31.90 1.94	0.0018** <0.0001*** 0.1032 ns	0.74 29.31 0.62	0.4760 ns <0.0001*** 0.6482 ns
LAC	100 10 0	113 105 113	0.40 a 0.33 b 0.33 b	10 10 10	140 133 133	0.40 a 0.42 a 0.46 a	N_S_ Day Interaction	8.39 10.01 2.64	0.0003*** <0.0001*** 0.0340*	1.79 2.93 0.32	0.1684 ns 0.0543 ns 0.8625 ns

LEGEND: N – number of patterns; N_S_ – number of succussion strokes; GLD – grey level distribution; FP – foreground pixels; LCFD – local connected fractal dimension; LAC – lacunarity; * – p < 0.05; ** – p < 0.01; *** – p < 0.001; ns – not significant.

**Table 2:** Results of pattern evaluation of *Baptisia* 10^−3^ samples prepared with different numbers of succussion strokes (N_S_ = 100, 10, or 0) and systematic positive control (SPC) experiments (on the left) and F-test of the two-way analysis of variance for the factors *N*_*S*_ and *day* (on the right). Mean values with different letter codes (a, b, c) are significantly different (p < 0.05).

	*Baptisia* 10^−3^			SPC			Factor	*Baptisia* 10^−3^		SPC	
	N_S_	N	Mean	N_S_	N	Mean		*F*	*p*	*F*	*p*
GLD	100 10 0	142 136 137	4.75 b 4.89 b 6.47 a	10 10 10	130 129 128	5.03 a 4.94 a 5.03 a	N_S_ Day Interaction	26.59 18.14 8.40	<0.0001*** <0.0001*** <0.0001***	0.08 88.83 4.35	0.9181 ns <0.0001*** 0.0019**
Entropy	100 10 0	142 136 137	1.90 b 1.85 b 1.97 a	10 10 10	130 129 128	2.56 a 2.43 b 2.60 a	N_S_ Day Interaction	4.34 28.92 5.15	0.0136* <0.0001*** 0.0005***	5.16 76.47 6.88	0.0062** <0.0001*** <0.0001***
FP	100 10 0	142 136 137	2.77 × 10^3^ b 2.92 × 10^3^ b 4.65 × 10^3^ a	10 10 10	130 129 128	3.05 × 10^3^ a 2.37 × 10^3^ b 2.59 × 10^3^ ab	N_S_ Day Interaction	15.72 6.51 7.44	<0.0001*** 0.0016** <0.0001***	3.24 27.09 6.11	0.0404* <0.0001*** 0.0001***
LCFD	100 10 0	142 136 137	1.11 b 1.09 b 1.43 a	10 10 10	130 129 128	0.88 a 0.86 ab 0.80 b	N_S_ Day Interaction	25.95 13.62 7.89	<0.0001*** <0.0001*** <0.0001***	2.62 183.83 10.41	0.0744 ns <0.0001*** <0.0001***
LAC	100 10 0	142 136 137	0.20 a 0.20 a 0.10 b	10 10 10	130 129 128	0.19 b 0.22 ab 0.23 a	N_S_ Day Interaction	37.28 23.10 7.80	<0.0001*** <0.0001*** <0.0001***	2.39 82.50 8.00	0.0927 ns <0.0001*** <0.0001***

LEGEND: N – number of patterns; N_S_ – number of succussion strokes; GLD – grey level distribution; FP – foreground pixels; LCFD – local connected fractal dimension; LAC – lacunarity; * – p < 0.05; ** – p < 0.01; *** – p < 0.001; ns – not significant.

**Table 3:** Results of pattern evaluation of *Baptisia* 10^−4^ samples prepared with different numbers of succussion strokes (N_S_ = 100, 10, or 0) and systematic positive control (SPC) experiments (on the left) and F-test of the two-way analysis of variance for the factors *N*_*S*_ and *day* (on the right). Mean values with different letter codes (a, b, c) are significantly different (p < 0.05).

	*Baptisia* 10^−4^			SPC			Factor	*Baptisia* 10^−4^		SPC	
	N_S_	N	Mean	N_S_	N	Mean		*F*	*p*	*F*	*p*
GLD	100 10 0	158 151 152	1.47 a 1.37 a 1.00 b	10 10 10	133 129 124	0.78 a 0.83 a 0.83 a	N_S_ Day Interaction	14.70 92.20 3.53	<0.0001*** <0.0001*** 0.0074**	0.30 274.39 7.91	0.7380 ns <0.0001*** <0.0001***
Entropy	100 10 0	158 151 152	1.82 a 1.78 a 1.62 b	10 10 10	133 129 124	1.49 a 1.49 a 1.45 a	N_S_ Day Interaction	12.88 173.60 2.47	<0.0001*** <0.0001*** 0.0438*	0.54 376.74 5.95	0.5810 ns < 0.0001*** 0.0001***
FP	100 10 0	158 151 152	1.60 × 104 a 1.27 × 10^4^ b 1.00 × 10^4^ c	10 10 10	133 129 124	1.34 × 104 a 1.61 × 104 a 0.89 × 104 b	N_S_ Day Interaction	8.65 47.37 4.41	0.0002*** <0.0001*** 0.0017**	5.20 43.85 5.80	0.0059** <0.0001*** 0.0002***
LCFD	100 10 0	158 151 152	1.20 b 1.20 b 1.27 a	10 10 10	133 129 124	1.30 a 1.31 a 1.35 a	N_S_ Day Interaction	3.41 38.52 3.74	0.0337* <0.0001*** 0.0053**	0.92 70.08 7.37	0.3995 ns <0.0001*** <0.0001***
LAC	100 10 0	158 151 152	0.30 a 0.30 a 0.25 b	10 10 10	133 129 124	0.25 a 0.24 a 0.24 a	N_S_ Day Interaction	5.56 28.43 3.33	0.0041** <0.0001*** 0.0106*	0.29 52.22 7.42	0.7456 ns <0.0001*** <0.0001***

LEGEND: N – number of patterns; N_S_ – number of succussion strokes; GLD – grey level distribution; FP – foreground pixels; LCFD – local connected fractal dimension; LAC – lacunarity; * – p < 0.05; ** – p < 0.01; *** – p < 0.001; ns – not significant.

**Table 4:** Results of pattern evaluation of *Luffa* 10^−4^ samples prepared with different numbers of succussion strokes (N_S_ = 100, 10, or 0) and systematic positive control (SPC) experiments (on the left) and F-test of the two-way analysis of variance for the factors *N*_*S*_ and *day* (on the right). Mean values with different letter codes (a, b, c) are significantly different (p < 0.05).

	*Luffa* 10^−4^			SPC			Factor	*Luffa* 10^−4^		SPC	
	N_S_	N	Mean	N_S_	N	Mean		*F*	*p*	*F*	*p*
GLD	100 10 0	136 152 122	5.37 b 5.39 b 6.49 a	10 10 10	143 133 137	5.41 a 5.80 a 5.44 a	N_S_ Day Interaction	6.88 8.73 10.76	0.0012** 0.0002*** <0.0001***	1.82 133.72 4.55	0.1630 ns <0.0001*** 0.0013**
Entropy	100 10 0	136 152 122	2.12 a 2.07 a 2.16 a	10 10 10	143 133 137	1.91 a 1.98 a 1.91 a	N_S_ Day Interaction	1.04 18.03 11.36	0.3537 ns <0.0001*** <0.0001***	1.80 52.24 1.78	0.1673 ns <0.0001*** 0.1328 ns
FP	100 10 0	136 152 122	2.64 × 10^3^ b 2.59 × 10^3^ b 3.49 × 10^3^ a	10 10 10	143 133 137	4.04 × 10^3^ ab 4.59 × 10^3^ a 3.82 × 10^3^ b	N_S_ Day Interaction	11.41 22.73 5.69	<0.0001*** <0.0001*** 0.0002***	1.71 18.70 0.37	0.1819 ns <0.0001*** 0.8297 ns
LCFD	100 10 0	136 152 122	0.66 b 0.67 b 0.86 a	10 10 10	143 133 137	0.93 a 0.93 a 0.86 a	N_S_ Day Interaction	19.59 1.84 4.06	<0.0001*** 0.1609 ns 0.0031**	1.28 41.14 1.62	0.2803 ns <0.0001*** 0.1690 ns
LAC	100 10 0	136 152 122	0.48 a 0.41 b 0.33 c	10 10 10	143 133 137	0.31 a 0.31 a 0.33 a	N_S_ Day Interaction	20.05 9.35 3.75	<0.0001*** 0.0001*** 0.0051**	0.68 40.43 1.10	0.5047 ns <0.0001*** 0.3577 ns

LEGEND: N – number of patterns; N_S_ – number of succussion strokes; GLD – grey level distribution; FP – foreground pixels; LCFD – local connected fractal dimension; LAC – lacunarity; * – p < 0.05; ** – p < 0.01; *** – p < 0.001; ns – not significant.

**Table 5:** Results of pattern evaluation of *Spongia* 10^−6^ samples prepared with different numbers of succussion strokes (N_S_ = 100, 10, or 0) and systematic positive control (SPC) experiments (on the left) and F-test of the two-way analysis of variance for the factors *N*_*S*_ and *day* (on the right). Mean values with different letter codes (a, b, c) are significantly different (p < 0.05).

	*Spongia* 10^−6^			SPC			Factor	*Spongia* 10^−6^		SPC	
	N_S_	N	Mean	N_S_	N	Mean		*F*	*p*	*F*	*p*
GLD	100 10 0	131 128 136	1.97 b 2.31 a 1.95 b	10 10 10	120 119 115	0.72 a 0.80 a 0.82 a	N_S_ Day Interaction	2.87 15.68 2.50	0.0574 ns <0.0001*** 0.0424*	1.23 556.16 2.44	0.2937 ns <0.0001*** 0.0469*
Entropy	100 10 0	131 128 136	1.77 a 1.83 a 1.68 b	10 10 10	120 119 115	1.03 a 1.02 a 0.98 a	N_S_ Day Interaction	7.12 21.56 7.05	0.0009*** <0.0001*** <0.0001***	1.38 259.24 6.19	0.2519 ns <0.0001*** 0.0001***
FP	100 10 0	131 128 136	3.42 × 10^4^ a 3.42 × 10^4^ a 3.44 × 10^4^ a	10 10 10	120 119 115	1.72 × 10^4^ a 1.71 × 10^4^ a 1.35 × 10^4^ b	N_S_ Day Interaction	0.002 3.23 3.02	0.9980 ns 0.0408* 0.0180*	1.84 138.33 2.90	0.1600 ns <0.0001*** 0.0219*
LCFD	100 10 0	131 128 136	1.54 b 1.47 c 1.60 a	10 10 10	120 119 115	1.48 a 1.47 a 1.47 a	N_S_ Day Interaction	13.71 16.65 1.35	<0.0001*** <0.0001*** 0.2498 ns	0.20 71.55 3.62	0.8208 ns <0.0001*** 0.0066**
LAC	100 10 0	131 128 136	0.16 b 0.19 a 0.14 b	10 10 10	120 119 115	0.18 a 0.20 a 0.18 a	N_S_ Day Interaction	11.11 15.28 1.44	<0.0001*** <0.0001*** 0.2207 ns	1.07 48.60 4.33	0.3447 ns <0.0001*** 0.0020**

LEGEND: N – number of patterns; N_S_ – number of succussion strokes; GLD – grey level distribution; FP – foreground pixels; LCFD – local connected fractal dimension; LAC – lacunarity; * – p < 0.05; ** – p < 0.01; *** – p < 0.001; ns – not significant.

### Echinacea 10^−2^

In case of *Echinacea* 10^−2^ (Table 1) application of succussion significantly increased the pattern evaluation parameters *grey level distribution* (GLD) and *entropy* (for N_S_ = 10, 100). Also, the fractality parameters *local connected fractal dimension* (LCFD) and *lacunarity* increased following the succussion, however, LCFD only for N_S_ = 10 and *lacunarity* only for N_S_ = 100.

All systematic control experiments performed did not show any significance between the randomization groups for the main effects.

### Baptisia 10^−3^

As shown in Table 2, *Baptisia* 10^−3^ succussed samples (N_S_ = 100, 10) were characterized by significantly lower GLD, *entropy*, FP, and LCFD values compared to the unsuccussed samples, whereas *lacunarity* was significantly higher.

The systematic control experiments yielded a significant main effect for the parameters FP and *entropy*; the other three image analysis parameters did not show statistically significant differences between the randomization groups for the main effects. Thus, the main experiments’ outcome regarding FP and *entropy* can be distorted due to chamber gradients (see below) and was excluded from further evaluation.

### Baptisia 10^−4^

In case of *Baptisia* 10^−4^ the parameter FP could differentiate significantly between all samples (N_S_ = 0, 10, 100); whereas the parameters GLD, *entropy, LCFD*, and *lacunarity* differentiated between the succussed (N_S_ = 10, 100) and unsuccussed (N_S_ = 0) samples (Table 3).

The systematic control experiments yielded a significant main effect for the parameter FP; the other four image analysis parameters did not show statistically significant differences between the randomization groups for the main effects. Thus, the main experiments’ outcome regarding FP can be distorted due to chamber gradients (see below) and was excluded from further evaluation.

### *Luffa* 10^−4^

For *Luffa* 10^−4^ GLD, FP, and LCFD decreased significantly in the succussed samples, whereas *lacunarity* increased (Table 4). Parameter *lacunarity* significantly differentiated all samples (N_S_ = 100, 10, and 0); whereas parameter *entropy* showed no significance between the samples in the main experiments.

No systematic control experiment performed showed significant main effects between the randomization groups.

### Spongia 10^−6^

In case of *Spongia* 10^−6^ (Table 5) parameter LCFD differentiated all samples and ranked them in the order N_S_ 0 > 100 > 10; whereas *lacunarity* yielded significantly higher values only for the sample N_S_ = 10. Parameter *entropy* differentiated the succussed samples (N_S_ = 100, 10) from the unsuccussed ones. The parameters GLD and FP did not differentiate the samples.

No systematic control experiment performed showed significant main effects between the randomization groups.

### Influence of succussion on DEM patterns

In order to summarize the experimental results, in Table 6 we considered as relevant only cases where the corresponding image analysis parameter was experimentally stable, which means that (i) the systematic positive control experiments were not significant, and (ii) in the F-test of analysis of variance of the main experiments the F value for the factor *N*_*S*_ was higher than the F value for the interaction *N*_*S*_ and *day*. This means that 16 out of 25 parameter/preparation combinations were retained.

**Table 6:** Graphical representation of relevant differences found in the image evaluation parameters in pharmaceutical preparations prepared with varying numbers of succussion strokes N_S_ = 100, 10, or 0. Different letters (a, b, c) indicate significant differences at *p* < 0.05.

	*Echinacea* 10^−2^			*Baptisia* 10^−3^			*Baptisia* 10^−4^			*Luffa* 10^−4^				*Spongia* 10^−6^			
GLD	100 10 0	a a	b	100 10 0	a	b b	100 10 0	a a	b								
Entropy	100 10 0	a a	b				100 10 0	a a	b					100 10 0	a a	b	
FP										100 10 0	a	b b					
LCFD	100 10 0	a	b b	100 10 0	a	b b				100 10 0	a	b b		100 10 0	a	b	c
LAC	100 10 0	a	b b	100 10 0	a a	b	100 10 0	a a	b	100 10 0	a	b	c	100 10 0	a	b b	

LEGEND: GLD – grey level distribution; FP – foreground pixels; LCFD – local connected fractal dimension; LAC – lacunarity.

Overall, we observed significant differences for at least one sample (N_S_ = 100 or 10) compared to N_S_ = 0 in all analyzed comparisons (100%, 16/16). In most cases (68.75% of comparisons, 11/16), the difference was between the succussed (N_S_ = 100, 10) and unsuccussed (N_S_ = 0) samples, without differentiating between the succussed samples. In 12.50% (2/16) of cases all samples (N_S_ = 100, 10, and 0) could be significantly differentiated; in 12.50% (2/16) of cases the N_S_ = 10 sample differed from the two others (N_S_ = 0, 100); and in one case (6.25%, 1/16) the N_S_ = 100 sample differed from the two others (N_S_ = 0, 10).

Generalizing, it can be said that the GLD did not show a general direction of the influence of the succussion on the patterns; whereas in patterns from the succussed samples the pattern evaluation parameter *entropy* increased, and LCFD decreased. *Lacunarity* was the unique parameter showing significant differences for all pharmaceutical preparations and in general showed increased values in the succussed samples. FP differentiated the samples only in case of one remedy (*Luffa* 10^−4^).

### Climatized chamber gradients

Results of the F-test of the two-way analysis of variance with independent factors *row* and *column* from the systematic positive control experiments performed with *Echinacea* 10^−2^, *Baptisia* 10^−3^, *Baptisia* 10^−4^, *Luffa* 10^−4^, and *Spongia* 10^−6^ are shown in Table 7. As it can be noticed, factor *row* showed significance for most image evaluation parameters of the patterns obtained from the five pharmaceutical preparations (14 results out of 25; 14/25), whereas factor *column* was significant only in one case (*Luffa* 10^−4^, parameter *lacunarity*). The interaction between factors *row* and *column* resulted also significant in 8/25 cases, however mostly with lower F values than those observed for factor *row*.

**Table 7:** F-test results of the analysis of variance with independent factors row and column of the systematic positive control experiments for *Echinacea* 10^−2^, *Baptisia* 10^−3^, *Baptisia* 10^−4^, *Luffa* 10^−4^, and *Spongia* 10^−6^ prepared by applying 10 succussion strokes in-between the dilution steps.

	Factor	*Echinacea* 10^−2^		*Baptisia* 10^−3^		*Baptisia* 10^−4^		*Luffa* 10^−4^		*Spongia* 10^−6^	
		*F*	*p*	*F*	*p*	*F*	*p*	*F*	*p*	*F*	*p*
GLD	Row Column Interaction	3.35 1.01 0.97	0.0191* 0.3644 ns 0.4443 ns	3.52 2.49 2.49	0.0152* 0.0839 ns 0.0223*	3.29 0.11 2.46	0.0208* 0.8910 ns 0.0238*	1.58 1.91 1.22	0.1934 ns 0.1496 ns 0.2968 ns	2.38 1.35 0.42	0.0691 ns 0.2592 ns 0.8625 ns
Entropy	Row Column Interaction	2.08 0.97 0.52	0.1019 ns 0.3787 ns 0.7958 ns	1.80 0.85 3.14	0.1457 ns 0.4269 ns 0.0051**	4.39 0.75 2.29	0.0047** 0.4737 ns 0.0351*	0.71 1.60 1.83	0.5460 ns 0.2039 ns 0.0920 ns	5.20 2.03 3.33	0.0016** 0.1324 ns 0.0034**
FP	Row Column Interaction	4.79 1.04 1.06	0.0027** 0.3532 ns 0.3839 ns	2.53 0.90 2.07	0.0570 ns 0.4053 ns 0.0564 ns	6.20 0.15 3.67	0.0004*** 0.8633 ns 0.0015**	0.75 0.21 0.51	0.5250 ns 0.8121 ns 0.7990 ns	0.88 1.05 0.59	0.4515 ns 0.3501 ns 0.7372 ns
LCFD	Row Column Interaction	2.95 1.24 1.55	0.0328* 0.2914 ns 0.1607 ns	2.34 1.47 1.47	0.0729 ns 0.2301 ns 0.1888 ns	5.15 1.08 3.50	0.0017** 0.3417 ns 0.0022**	2.65 1.54 1.08	0.0485* 0.2148 ns 0.3757 ns	0.15 1.46 1.39	0.9315 ns 0.2335 ns 0.2128 ns
LAC	Row Column Interaction	0.35 8.13 0.77	<0.0001*** 0.7018 ns 0.5902 ns	3.95 2.09 2.03	0.0086** 0.1245 ns 0.0605 ns	5.69 1.44 2.22	0.0008*** 0.2371 ns 0.0403*	3.90 3.69 0.24	0.0091** 0.0258* 0.9640 ns	0.34 1.37 2.01	0.7994 ns 0.2549 ns 0.0639 ns

LEGEND: GLD – grey level distribution; FP – foreground pixels; LCFD – local connected fractal dimension; LAC – lacunarity; * – p < 0.05; ** – p < 0.01; *** – p < 0.001; ns – not significant.

The quasi-randomization design applied in the differentiating experiments could eliminate the significant influence of chamber gradients (Table 7) in total in 13/14 cases and in the 16 retained experiments in 11/11 cases (Tables 1–5).

## Discussion

The results of the present study show that in all five analyzed pharmaceutical preparations the succussion strokes applied during production significantly influenced the DEM patterns. It can be summarized that succussion induced the formation of structures characterized by a greater disorder (parameter *entropy*) and smaller complexity (parameter *local connected fractal dimension*), at the same time increasing the gaps between the structure elements (parameter *lacunarity*). In case of two preparations (*Luffa* 10^−4^ and *Spongia* 10^−6^), significant differences could be found between all samples (N_S_ = 0, 10, and 100). The here chosen parameters have already been applied in structure analysis of patterns formed in course of phase transition of liquid pharmaceutical preparations^11^; moreover, raw material surfaces present in pharmaceutical triturations were also analyzed by means of fractal dimension^18^.

DEM patterns in the here analyzed dilution range 10^−2^-10^−6^ are in a first place a function of solute dry residue. Differences found between the patterns of succussed vs. not succussed samples might be linked with succussion-induced aggregation of large-size molecules^2,5^, or, in case of *Spongia* 10^−6^ (consisting only of mineral substances, since the sponge is roasted) through the introduction of air bubbles and/or particle formation^2^.

Whereas the patterns of *Echinacea* 10^−2^, *Baptisia* 10^−3^, *Luffa* 10^−4^, and *Spongia* 10^−6^ were concentrated in the central part of the droplet residue and fitted entirely on the photographed in 100× image, in case of *Baptisia* 10^−4^ the structures were rather unspecific and distributed almost evenly through the entire droplet residue (Fig. 1). In order to keep the magnification equal in the whole experimentation series, the part to be photographed was chosen by the experimenter (based on a visual check of the pattern, the part with most evident structures was chosen). The *Baptisia* 10^−4^ results might therefore be burdened with a certain bias; which, in future experimentations, might be overcome by adapting the experimental model.

The analysis of the systematic positive control experiments by the F-test of analysis of variance with independent factors *row* and *column* put in evidence that the factor *row* significantly influenced 14/25 parameters (Table 7). In most cases (13/14) this systematic error could be successfully eliminated (Tables 1–5) by the application of a quasi-randomization design, consisting in the randomization of the samples only within the columns, keeping simultaneously an even distribution of the samples within the rows. In future experiments, however, a better isolation of the inner-chamber should be aimed at to improve the homogeneity of evaporating conditions.

The influence of the factor *day* was significant in most of the here presented experiments (24/25 differentiation and 23/25 control experiments) (Tables 1–5). A significant influence of the experimentation day has been reported in many previous studies concerning methods based on phase-transition-induced pattern formation^11,13,14,19–22^. This fact might be due to some day-to-day variations in the experiment performance or experimental conditions; or to other yet unknown and uncontrolled influences.

To conclude, we observed that the application of the droplet evaporation method on pharmaceutical preparations led to the creation of patterns revealing differences for the parameters grey level distribution, texture, and fractality, dependent on the application of succussion and the number of succussion strokes performed during the pharmaceutical processing.

In the present investigation we performed succussion by shaking the cylinder with the solution by hand freely in the air, with the cylinder being filled to about 2/3 of its capacity. This kind of succussion is a usual procedure applied by many producers of pharmaceutical preparations, however it is not completely standardized and might vary in velocity and dynamic when performed by different persons. Further DEM experiments should be conducted comparing the impact of different methods of succussion, considering besides the quantity of performed movements also their intensity and type of movement.

The here presented experimental protocol might constitute a fairly economic and quick tool to investigate the impact of agitation on solutions, which has great importance for fabrication and distribution of pharmaceutical preparations in general and which is addressed in many recent investigations. In particular, it might serve to compare the role of several factors known for being critical for the solution properties, like for instance the kind of induced flow (e.g. chaotic vs. ordered, vortex-like)^2,3,23^, different surfaces and coatings of the recipient’s walls^24,25^, and different volumes of the headspace^2,26^. DEM might be applied alternatively or complementary to established analytical methods used for the characterization of succussed solutions, such as, *inter alia*, micro-flow imaging, dynamic light scattering, light obscuration method (serving for analyzing the formation of particles), size exclusion chromatography, and tryptic digestion/HPLC (for studying the aggregation of proteins), hydroxyphenyl fluorescein assay (analyzing the formation of free radicals), and fluorescence spectroscopy, Fourier transform infrared spectroscopy and differential scanning calorimetry (characterizing further the solution composition and thermodynamic characteristics)^1–6^. Comparison studies of DEM with these methods should be conducted to characterize the DEM patterns better in terms of specific solution properties. Furthermore, investigations on a possible link between the patterns and biological efficacy are needed.

## Methods

### Manufacturing of pharmaceutical preparations in dilution 10^−1^

*Echinacea* 10^−1^, *Baptisia* 10^−1^, *Luffa* 10^−1^, and *Spongia* 10^−1^ were manufactured by Hevert-Arzneimittel GmbH & Co. KG (Nussbaum, Germany) according to the European Pharmacopoeia, Homoeopathic Preparations^12^. In particular, *Baptisia* 10^−1^ and *Echinacea* 10^−1^ were prepared with the method 1.1.5 (i.e. first dilution in ratio 3:7), *Luffa* 10^−1^ with method 1.1.8 (i.e. first dilution in ratio 1:9), and *Spongia* 10^−1^ with method 1.1.9 (i.e. first dilution in ratio 2:8).

### Study design

**Figure 2:**
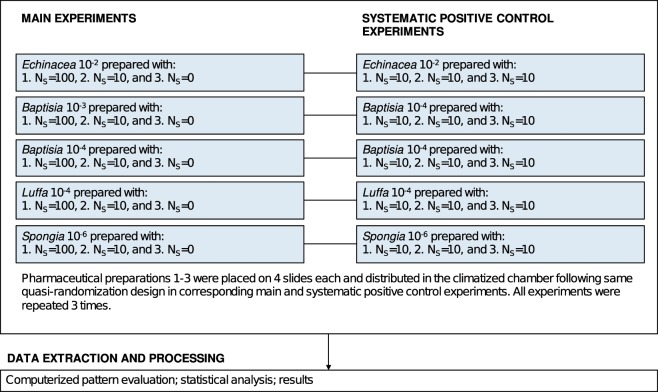
Flow-chart depicting the study design consisting of main experiments aiming at the differentiation of five pharmaceutical preparations (*Echinacea* 10^−2^, *Baptisia* 10^−3^, *Baptisia* 10^−4^, *Luffa* 10^−4^, and *Spongia* 10^−6^) prepared with different numbers of succussion strokes (N_S_ = 100, 10, or 0) and corresponding systematic positive control experiments.

**Figure 3:**
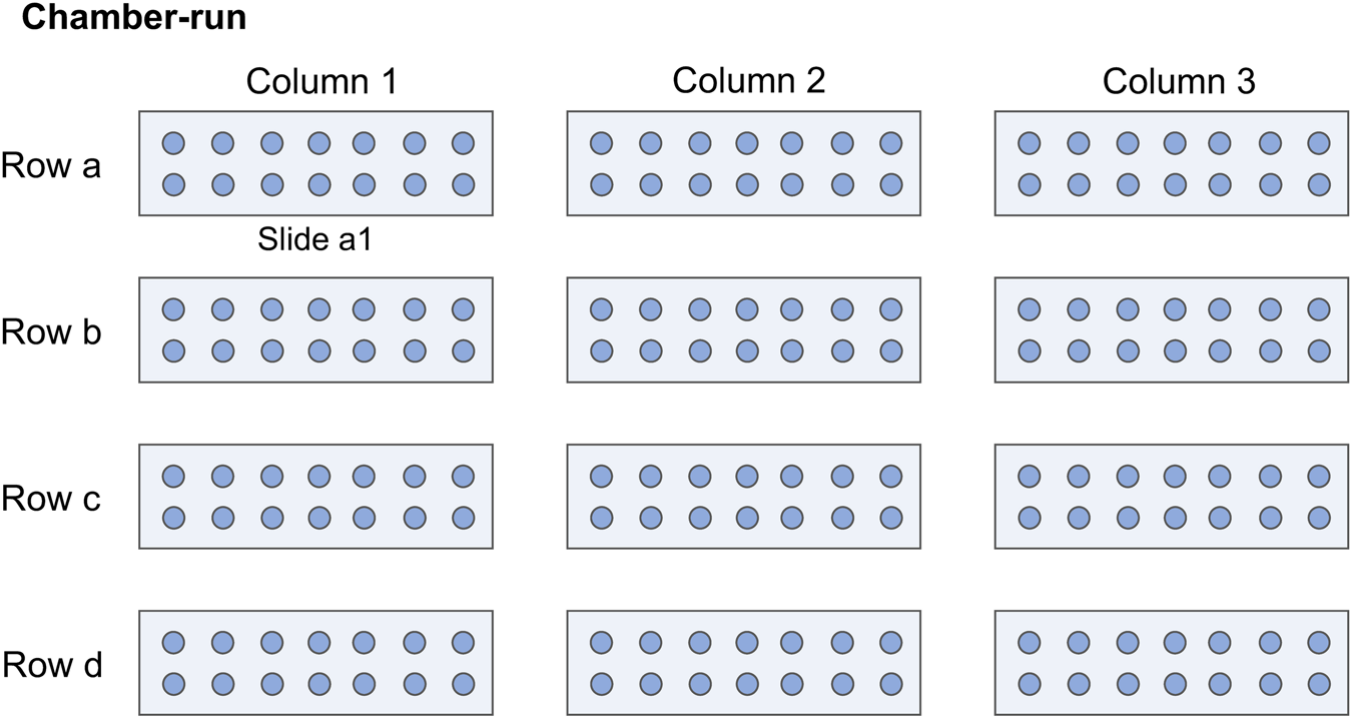
Graphical representation of one chamber-run design. The arrangement of the slides in the evaporation chamber was organized in 4 rows (a-d) and 3 columns (1–3), in which 12 slides were placed (slide a1, a2, … d3). On each slide 14 droplets were deposited for evaporation.

The experimentation took place in the laboratories of Society for Cancer Research (Arlesheim, Switzerland). As shown in Fig. 2 the study consisted of main experiments and full systematic positive control experiments. The main experiments were performed on five pharmaceutical preparations (*Echinacea* 10^−2^, *Baptisia* 10^−3^, *Baptisia* 10^−4^, *Luffa* 10^−4^, and *Spongia* 10^−6^), prepared from the 10^−1^ dilutions by applying different numbers of succussion strokes (N_S_ = 100, 10, or 0). These three variations of a given homeopathic preparation were analyzed in one experimental run, consisting of twelve slides with droplets deposited on them (Fig. 3). Four slides were used for each pharmaceutical preparation. The slides were distributed in a climatized chamber following a quasi-randomization design. Each main experiment had a corresponding systematic positive control experiment where the analyzed sample was prepared three times with N_S_ = 10 and analyzed following the same quasi-randomization design as in the main experiment. All experiments were independently repeated three times.

### Preparation of pharmaceutical preparations for analysis

0.8 g of a pharmaceutical preparation in dilution 10^−1^ was weighed and placed in a sterile glass cylinder (SBR-ET, Mix Cyl. 10 ml, B; Brand GmbH + CO KG, Wertheim, Germany) with stopper (untargeted volume 13 ml); subsequently 7.2 ml purified water according to Pharm. Eur. 9.4^12^ (“purified water in bulk”, X-SEPTRON LINE 10 VAL, BWT AQUA AG, Aesch, Switzerland) was added in order to reach a dilution of 1:9. The cylinder was closed tightly; 10 or 100 succussion strokes were applied by hand. The movement to achieve succussion was performed in the air without hitting against a firm base. For the unsuccussed samples, the content of the cylinder was mixed with a glass stirrer by performing circular movements in order to not create any foam. After the settling of any foam in preparations N_S_ = 10 and 100, the cylinders were re-opened and 0.8 ml of the solution were taken for the preparation of the next dilution, as described previously. In this way three variants (N_S_ = 100, 10, 0) of each preparation (*Echinacea* 10^−2^, *Baptisia* 10^−3^, *Baptisia* 10^−4^, *Luffa* 10^−4^, and *Spongia* 10^−6^) were produced. All samples were prepared fresh for each experiment. The samples were not blinded.

### Droplet evaporation method

Microscope slides (76 × 26 mm, pre-cleaned, cut edges; Thermo Scientific, Gerhard Menzel B.V. & Co. KG, Braunschweig, Germany) were degreased by washing them with a dishwasher liquid, then thoroughly rinsed with hot tap water, and placed in 4 consecutive purified water baths. Each slide was wiped dry with a laboratory wiper (KIMTECH science, Kimberly-Clark Professional, Roswell, Canada) just before droplet deposition. 3 μl droplets of the tested pharmaceutical preparation were deposited on the slides in two parallel rows, 7 droplets per row, by the use of a micro-pipette of 20 µl capacity (Eppendorf Research Plus, Eppendorf, Hamburg, Germany).

Evaporation took place in an incubator (KBF 720, cooled incubator with controlled humidity system, WTB Binder Labortechnik GmbH, Tuttingen, Germany) with an inner plexi-glass-chamber with a semi-permeable cover placed on a vibration absorbing basis. The microscope slides with droplets were placed in the inner-chamber and left for evaporation in 26 °C and 44%rH for 1 hour. The slide distribution inside the chamber followed a quasi-randomization design in order to provide a uniform arrangement of the samples within the rows (Fig. 2).

### Photographing of patterns

The droplet residues were examined and photographed in dark field in magnification 100× by use of an optical microscope (Zeiss Lab.A1; Carl Zeiss Microscopy GmbH, Jena, Germany) with an attached camera (Moticam 5.0 MP; CMOS; Motic Electric Group Co., Ltd, Xiamen, China). Droplets with disturbed crystallization due to presence of contaminating particles or due to edge effects on the slide were not considered. Per experiment (one chamber-run, Fig. 3), 168 droplets were prepared (14 droplets x 12 slides).

For *Echinacea* 10^−2^ the three main experiments yielded 399 evaluable droplet residue images and the three positive control experiments 406 images (399/406); for *Baptisia* 10^−3^ 415/387; for *Baptisia* 10^−4^ 461/386; for *Luffa* 10^−4^ 410/413; and for *Spongia* 10^−6^ 395/354, giving in total 4’026 images. Images were saved in jpeg-format (2592 × 1944 pixel).

In case of *Echinacea* 10^−2^, *Baptisia* 10^−3^, *Luffa* 10^−4^, and *Spongia* 10^−6^, the 100X images included the whole structure formed inside the droplet; whereas, in case of *Baptisia* 10^−4^, only selected parts of the structure were included, chosen by the experimenter on the basis of density and intensity of forms.

### Computerized pattern evaluation

Image analysis was performed with the software ImageJ (v. 1.50b)^27^ with the plug-ins *GLCM Texture*^28^ and *Frac-Lac*^16^. All 100× images were subjected to a background extraction by means of the sliding paraboloid with rolling ball radius set at 50 pixels ensuring same background throughout the image database. Consecutively the images were analyzed (i) for their grey-level distribution, (ii) after conversion into 8-bit type, by running the GLCM algorithm (considering distances between pixel pairs of 4 pixels and angles of 90°), for their texture (parameter *entropy*), and (iii) after conversion into binary, by means of *Frac-Lac’s DLC* tool with odd sizes scaling method and size limits for the grid caliber series of minimum 4 and maximum 40 pixels, for the size of the structures (parameter *foreground pixels)*, complexity (parameter *local connected fractal dimension*), and characterization of the gaps between the structure elements (parameter *lacunarity*). After conversion into binary, 68 *Echinacea* 10^−2^ images could not be used due to a too dense ramification-network, and were excluded from fractality analysis. Whereas, in case of *Baptisia* 10^−3^ and *Luffa* 10^−4^, fractal analysis was performed on images reduced in size to 500 × 375 pixel.

### Statistical analysis

The data deriving from the computerized image analysis were analyzed by means of a two-way analysis of variance (CoStat, v. 6.311) (CoHort Software, Monterey, USA) at alpha = 0.05 with independent factors *number of succussion strokes* (N_S_) and *day* or *row* and *column*. An interaction term between the independent factors was included in the statistical model in order to assess stability and reproducibility. Distribution of data was checked by visual inspection. Slight deviations from normality were irrelevant due to the central limit theorem. Data-sets with larger deviations from normality were logarithmically transformed (log10); in total 18 data sets were transformed (*Echinacea* 10^−2^ main/control study: FP, LAC/FP, LAC; *Baptisia* 10^−3^: FP, LAC/FP, LAC; *Baptisia* 10^−4^: GLD, FP/GLD, FP; *Luffa* 10^−4^: FP/FP; *Spongia* 10^−6^: GLD, FP/GLD, FP). Global significance was determined with F-tests. Pairwise mean comparison was performed two-tailed with the protected Fisher’s least significant difference test (pairwise comparisons were evaluated only if the global F-test was significant at p < 0.05). This procedure gives a good safeguard against type I as well as type II errors, and thus balances well between false-positive and false-negative conclusions^29^. Results of the transformed data sets were back-transformed for presentation.

## Data Availability

The datasets generated and analyzed during the current study are available from the corresponding author on reasonable request.
